# Investigation of availability of a high throughput screening method for predicting butanol solvent -producing ability of *Clostridium beijerinckii*

**DOI:** 10.1186/s12866-016-0776-6

**Published:** 2016-07-22

**Authors:** HaiFeng Su, Jun Zhu, Gang Liu, Furong Tan

**Affiliations:** Enviromentally-Begnin Chemical Process Research Center, Division of Ecological & Enviromental Research on the Three Gorges, Chongqing Institute of Green and Interligent Technology, Chinese Academy of Science, Beijing, China; Biogas Institute of Ministry of Agriculture, Chengdu, 610041 Sichuan Peoples Republic of China; Sichuan Academy of Grassland Science, Xipu Chengdu, 611731 Sichuan, Peoples Republic of China; Rice Research Institute, Sichuan Agricultural University, 611130 Wenjiang, Sichuan China

**Keywords:** Screening, *Clostridium beijerinckii*, Butanol, Trypan blue dye, Starch

## Abstract

**Background:**

Currently, efficient screening methods for selection of desired bacterial phenotypes from large populations are not easy feasible or readily available due to the complicated physiological and metabolic networks of solventogenic clostridia. In this study, to contribute to the improvement of methods for predicting the butanol-producing ability of *Clostridium beijerinckii* based on starch substrate, we further investigate a simple, visualization screening method for selecting target strains from mutant library of *Clostridium beijerinckii* NCIMB 8052 by using trypan blue dye as an indicator in solid starch via statistical survey and validation of fermentation experiment with controlling pH.

**Results:**

To verify an effective, efficient phenotypic screening method for isolating high butanol-producing mutants, the revalidation process was conducted based on Trypan Blue was used for visualization, and starch was used as the bacterial metabolic substrate. The availability of the screening system was further evaluated based on the relationship between characteristics of mutant strains and their α-amylase activities. Mutant clones were analyzed in detail based on their distinctive growth patterns and rate of fermentation of soluble starch to form butanol and were compared by statistical method. Significant correlations were identified between colony morphology and changes in butanol concentrations. The screening method was validated via statistical analysis for characterizing phenotypic parameters. The fermentation experiment of mutant strains with controlling pH value also demonstrated a positive correlation between increased α-amylase activity and increased solvent production by *Clostridium beijerinckii* was observed, and therefore indicated that the trypan blue dyeing method can be used as a fast method to screen target mutant strain for better solvent producers from, for instance, a mutant library.

**Conclusions:**

The suitability of the novel screening procedure was validated, opening up a new indicator of approach to select mutant solventogenic clostridia with improved fermentation of starch to increase butanol concentrations. The applicability can easily be broadened to a wide range of interesting microbes such as cellulolytic or acetogenic microorganisms, which produce biofuels from feedstock rich in starch.

**Electronic supplementary material:**

The online version of this article (doi:10.1186/s12866-016-0776-6) contains supplementary material, which is available to authorized users.

## Background

With the development of environmental protection and sustainability, many countries develop alternative bioenergy sources in a planned way. One well-known example is fermentation of starch crops by microbes to generate acetone, butanol, and ethanol (ABE fermentation) [1–3]. Solventogenic *Clostridium* species *Clostridium beijerinckii* is used for biofuel production due to its unique ability to produce butanol via fermentation [4–7]. In order to produce *C. beijerinckii* strains suitable for industrial applications, it is necessary to generate an efficient mutant strain using metabolic engineering or classic chemical mutagenesis.

Great progress has been made in the industrial manufacture of butanol due to the use of modern biotechnology tools for genetic improvement of microorganisms for industrial use, including metabolic engineering and protein-directed evolution, with target genes deduced from transcriptome analyses or simply by common sense. However, metabolic engineering in *C. beijerinckii* is hindered due to the complex genetic background of *Clostridium*, obtaining a strain with high yield through genetic modification of the metabolic pathways of wild-type strains has proven challenging. Some researchers directed the metabolic flow toward butanol production by knocking out related genes in other metabolic pathways. In recent years, there have been many initiatives to increase butanol yields by transforming *C. acetobutylicum* and *C. beijerinckii* with genetic engineering and other tools. Previous studies have focused on increasing the metabolic flux toward the desired product of butanol by inactivating competing pathways. For example, targeting the acetone biosynthetic pathway and reducing or eliminating the unwanted byproducts; however, through this method, the butanol titer was also reduced compared to the wild-type strain [8–10]. Besides these efforts with *C. acetobutylicum*, there have no reports that high butanol yield was obtained through breeding with the *C. beijerinckii* strain to directly ferment starch crops in industrial applications. This strategy reduced shunt of metabolic precursors from competing metabolic pathways. It has been shown that if the acetone biosynthetic pathway is reduced, or unwanted by-products are eliminated, butanol production decreases [10–12]. These results underscore the difficulty of manipulating metabolic pathways of *C. beijerinckii* to obtain a strain with high butanol yield. The transformation of the Clostridium metabolic pathway via metabolic engineering approaches to obtain mutant strains that produce high yields of butanol has been very difficult due to the fact that solventogenic clostridia has complicated physiological and metabolic networks. Therefore, traditional methods, such as classic chemical mutagenesis, are still an option for an effective approach to obtain desired strains capable of producing high yields of butanol.

Although classic chemical mutagenesis has been demonstrated potential to create strains of Clostridium with improved fermentation performance for obtaining high yields of butanol, the typical phenotype of a strain with high butanol titer is difficult to determine due to a large number of unknown parameters, therefore the identification of a quick, feasible method to identify mutant strains for specific parameters via screening is particularly important. Even with classic chemical mutagenesis, it is difficult to isolate a mutant strain of *C. beijerinckii* capable of producing high amounts of butanol from a large population of mutants with unknown genotypes. Isolation of a target strain for industrial use is performed by analyzing a large number of phenotypic parameters, and is laborious and time-consuming.

Some high-throughput screening methods have been developed for screening target mutant strains. One screening method is based on the “tolerance of strains”. In another method, mutant populations are exposed to suicide substrates like allyl alcohol and bromobutyrate to select for solvent-negative mutants [13, 14]. More recently, flow cytometry has been employed to analyze *C. acetobutylicum* and *Clostridium pasteurianum* mutant cellular morphology [15–17]. The most frequently employed screening technique to isolate target strains requires measuring butanol concentration and selecting for butanol-tolerant strains [18]. This method is correlated with increased butanol production in mutant strains, but can select for strains without increased butanol capacities [19]. The strain *C. acetobutylicum* ATCC 10132 was isolated after mutagenic treatment with bromide and bromate as selective agents. The mutants altered in acetic acid synthesis or demonstrated a shift to solventogenesis were selected directly via a proton suicide method. In the selection plates, mutants differed in colony phenotype from the parent strains [20]. Another technique has been developed for predicting the solvent-producing ability of *C. acetobutylicum*. Using this technique, the relationship between colony morphology and solvent production provides a method for predicting the solvent-producing potential of the cultures [21]. Using glucose pyrophosphorylase and granulose synthase as indices, Reysenbach et al*.* found that mutants that demonstrated granulose accumulation lacked either one or both enzyme activities necessary for its metabolism [22]. Another screening method is based on α-amylase activity. This method makes use of changes in regulation and localization of amylolytic enzymes in *C. acetobutylicum* by mutagenesis [23]. Finally, semi or high-throughput screening can be used to identify mutant strains. Using a transcription factor-based biosensor for specific activation, strains were screened for enhanced production of succinate, adipate, or 1-butanol [24]. Similarly, a high-throughput screening system identified a potentially useful strain of *C. acetobutylicum* [25]. Although there have been important developments in optimizing screening methods following chemical mutagenesis, obtaining mutant strains with high butanol yields still needs much work in progress. Although strain tolerance was useful for identification of certain mutants, is limited because the method does not necessarily relate to increased production capacities, and there are no reports to date that have used this method to obtain high yield strains. Screening mutants based on the activity of α-amylase is feasible, and high yield strains such as *C. beijerinckii* BA101 have been identified using this method. However, this method is not always consistent and can be unpredictable, so identifying a feasible way to detect α-amylase activity from a large number of mutant strains requires additional work. Similarly, high-throughput screening is a good method for detection of mutants. However, this technique requires expensive diagnostic reagent kits in the screening process, making it the most costly approach.

Thus it can be seen that using all of these methods still difficult to obtain target mutant strains. Therefore, the key challenge has been the identification of an efficient and simple method to identify and separate target strains with the desired phenotypes from a particular population independently from the respective genotypes. In general, the genetic perturbations are initially unknown. Therefore, devising such a screening method would represent a significance advance in this field of research. It is important to select an appropriate screening method to obtain the target mutants with the highest butanol yields.

It is necessary to refine a simple, efficient method in order to enable selection for high butanol yield. We aimed to develop an effective, efficient phenotypic screen for isolating high butanol-producing mutants. The procedure utilized Trypan Blue for visualization, and starch as the bacterial metabolic substrate. Starch is the most relevant substrate because starchy crops are the common fermentation substrates used in industrial settings. Trypan Blue is a dye used to stain cells; it is most often used for detecting cell membrane integrity and cellular survival. Vibrant cells are not stained by Trypan Blue, but cells in vitality decline are stained pale blue. There are numerous studies in the literature using Trypan Blue stain to assay cell growth [26–29]. Therefore, a screening method have been developed based on the characteristic and mechanism of Trypan Blue using solid starch medium as a metabolic substrate in these assays and Trypan Blue dye as an indicator by our previous study [30]. However, the screening method was just simply presented, lack of full detailed argument, its feasibility in other different fermentation substrates did not widely confirmed.

Here, the objective of this study was to investigate the simple, efficient phenotypic screening method for selecting *C. beijerinckii* strains with high butanol production via statistical survey and validation of fermentation experiment with controlling pH. The availability of screening method was further validated via statistical analysis for characterizing phenotypic parameters from a total of 76 clones in this study. Mutants with significantly raised butanol concentrations based on fermentation of soluble starch substrate were compared. The applicability of this method for strain-specific butanol production was demonstrated using mutant libraries generated by chemical mutagenesis via fermentation experiment with controlling pH.

## Methods

### Bacterial strains and cultivation conditions

The strain *C. beijerinckii* NCIMB 8052 from the National Collections of Industrial, Food and Marine Bacterial Center (NCIMB Ltd, UK) was used as starting strain of mutagenesis. The propagation and rejuvenation process of this microorganism were prepared, as described in the literature [30].

#### *Chemical mutagenesis*, *enrichment and isolation of mutants*

Chemical mutagenesis, enrichment process for cells of wild-type *C. beijerinckii* NCIMB 8052 was conducted according to the presentation in the literature [30]. The process of obtaining and isolateing of mutants have also been illustrated as requestment of the literature [30]. After colonies had formed on the plate, those with obvious clear zones were selected as mutants for use in fermentation experiments.

#### α-amylase activity assay and inhibition

To investigate the effect of amylase activity on butanol production, and to confirm the identity of the key enzyme, α-amylase activity assay and inhibition experiment was conducted, the experiment process was illustrated, as explained in the methods [30], the soluble starch was used as identification substrate via fermentation experiment.

### Fermentation processes with controlling pH

To prepare the fermentation inoculum for the cultivation of mutant strains, 100 mL TGYM medium containing 20 g/L glucose, 5 g/L yeast extract, 3 g/L ammonium acetate, 1 g/L sodium chloride, 1 g/L KH2PO4, 1 g/L K2HPO4, 0.2 g/L MgSO4, 0.02 g/L MnSO4 7H2O, and 0.02 g/L FeSO4 7H2O was autoclaved at 115 °C for 20 min. After the medium was cooled to 35 °C, 0.5 g/L cysteine was filter sterilized (0.22 μm Milliporefilter, GEMA Medical SL, Chengdu) and added to the medium. Approximately 5 mL of the prepared rejuvenation bacterial culture were then added to the medium, and the bacteria grew at 37 °C until the OD(600) = 1.5.

Glucose (60 g/L) and soluble starch (60 g/L) were used as fermentation substrates. Soluble starch was gelatinized at 121 °C for 1 h, and then cooled to room temperature for use in fermentation assays. Batch cultures were carried out statically. Fermentation bottles were sparged with filtered oxygen-free nitrogen gas to maintain strictly anaerobic conditions. The fermentation cultures were maintained anaerobically, and the pH was automatically maintained at 5.5 by a pH controller for 24 h (PHC-2201; Able, Tokyo, Japan). The fermentation processes were conducted in 500 mL glass anaerobic bottles (Haimen Huakai experiment glass instrument Co., Ltd, Haimen, China) sealed with butyl rubber. After addition of 1 g yeast extract and 2 g peptone to each bottle, the pH of the media was adjusted to 6.8 with 1 % NaOH immediately after addition of the fermentation substrates. The fermentation solutions were sterilized at 115 °C for 20 min, and cooled to room temperature. Then, 5 mL of a combination of trace elements mixture solution (50 g/L KH_2_PO_4_, 50 g/L K_2_HPO_4_, and 220 g/L CH_3_COONH_4_), vitamins (0.1 g/L para-aminobenzoic acid, 0.1 g/L thiamin, and 0.001 g/L biotin), and minerals (20 g/L MgSO_4_ 7H_2_O, 1 g/L MnSO_4_ H_2_O, 1 g/L FeSO_4_ 7H_2_O, and 1 g/L NaCl) were filter sterilized (Millipore filter; 0.22 μm) and added to each bottle. The bottles were then inoculated with 3 mL of fermentation inoculum (OD_600_ = 1.5).

### Quantification of fermentation products

The compounds (acetone, butanol, and ethanol) were measured with a gas chromatograph (GC) equipped with a flame ionization detector. The system was a model 6890 GC (Agilent Technologies, Santa Clara, CA, USA) with a model 7673A automatic injector, sampler, and controller (Hewlett-Packard). Alcohol compounds were separated using a ZB-WAX capillary column (30 m, 0.25 mm inside diameter, 0.25 μm film thickness; Phenomenex Inc., PA, USA). The GC oven temperature was held initially at 40 °C for 5 min, and then raised stepwise, by 15 °C/min, until it reached 150 °C. It was then raised by 50 °C/min up to 250 °C, and held for 4 min. Helium was used as the carrier gas, with an inlet pressure of 0.065 Mpa. The injector and detector were maintained at 220 °C. A 1 μL volume of supernatant from the culture broth was injected in split-injection mode at a 1:30 split ratio. Isobutanol was used as the internal standard.

### Statistical analysis

For each experiment and assay, we calculated the mean response variables and the standard deviations (SD), unless otherwise indicated. Comparisons of variable(s) were made with Student’s *t*-test, and *P* < 0.05 was considered to be significantly different. Statistical analyses were conducted using the software program SPSS 21.0 (IBM, USA).

## Results

### Mutant strain’s morphological characteristics

**Fig. 1:**
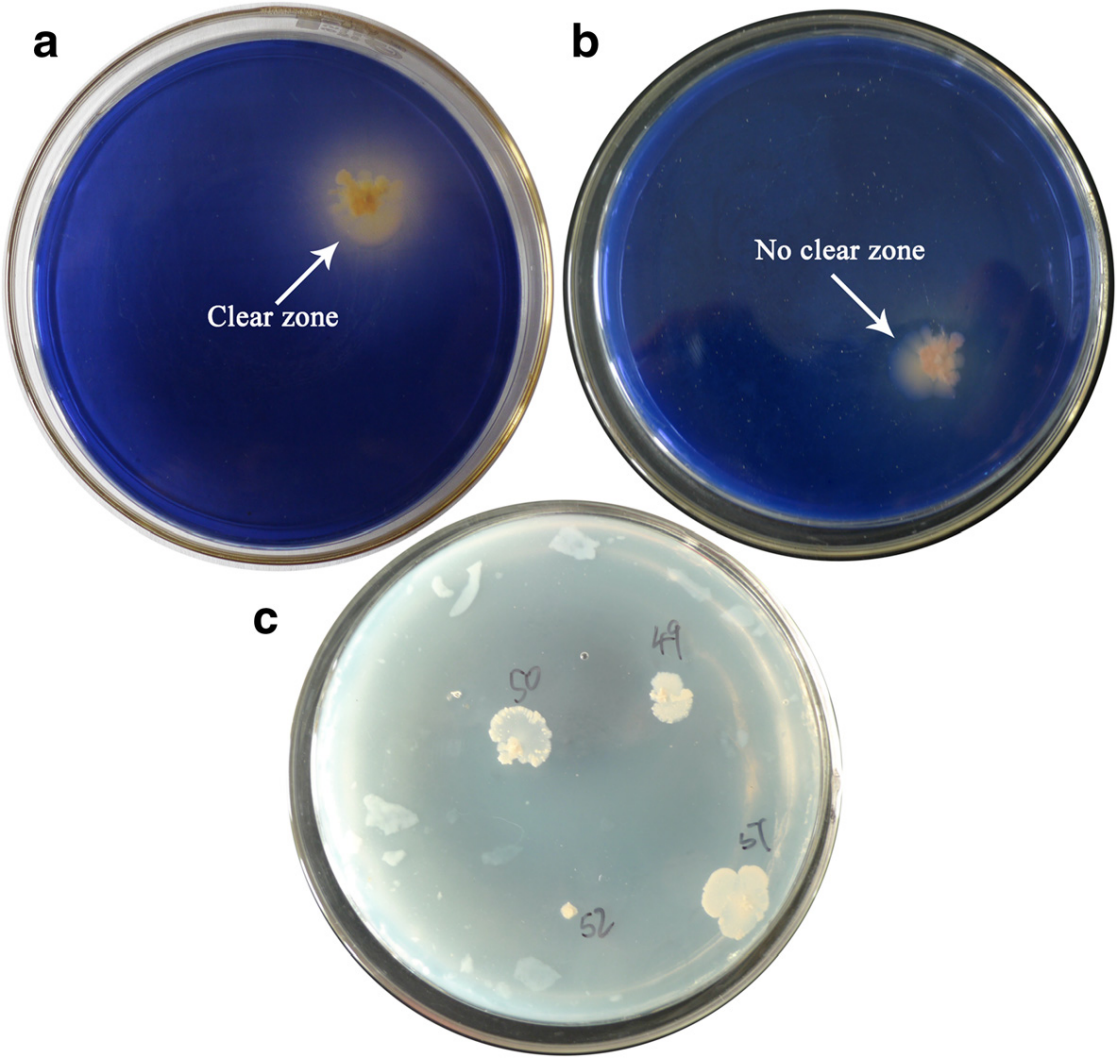
Mutant strains’ morphological characteristics in starch medium. **a** The mutant strains produced clear zone in the media containing trypan blue dye; **b** Some mutant strains did not produce clear zone in the media containing trypan blue dye. without clear zone; **c** The media without trypan blue dye

After mutagenesis, the bacterial suspension liquid was plated on STTY medium (50 μL bacteria liquid for each medium) and put in constant temperature anaerobic cultivation for 4 days until colonies became apparent. The phenotypic characteristics are presented in Fig. 1 (see Fig. 5b of Ref. [30]). The strain produce clear zone with brightness around the colony (Fig. 1a). There was even colony that did not produce a clear zone (Fig. 1b or see Ref. [30]). In addition, previous reports have presented the results showed that different mutant strains had different colony areas (see Fig. 5e, f and g of Ref. [30]), with differing diameter sizes and brightness around the colonies (see Fig. 5e and f of Ref. [30]).

Colonies showed significantly different characteristics when grown in culture medium with or without trypan blue (Fig. 1c). These remarkable features indicate that different mutant strains generated from the wild-type strain following chemical mutagenesis STTY may present different physiological characteristics when grown in STTY medium. Since the associated carbon source of the culture medium was starch, the morphological specificity of the different mutant stains may be related to changes in their α-amylase activity. A total of 76 colonies grew, and their regularity analysis such as distribution regularities was conducted by statistical survey.

### Relationships between butanol production and diameters of clear zone and α-amylase activity

**Fig. 2:**
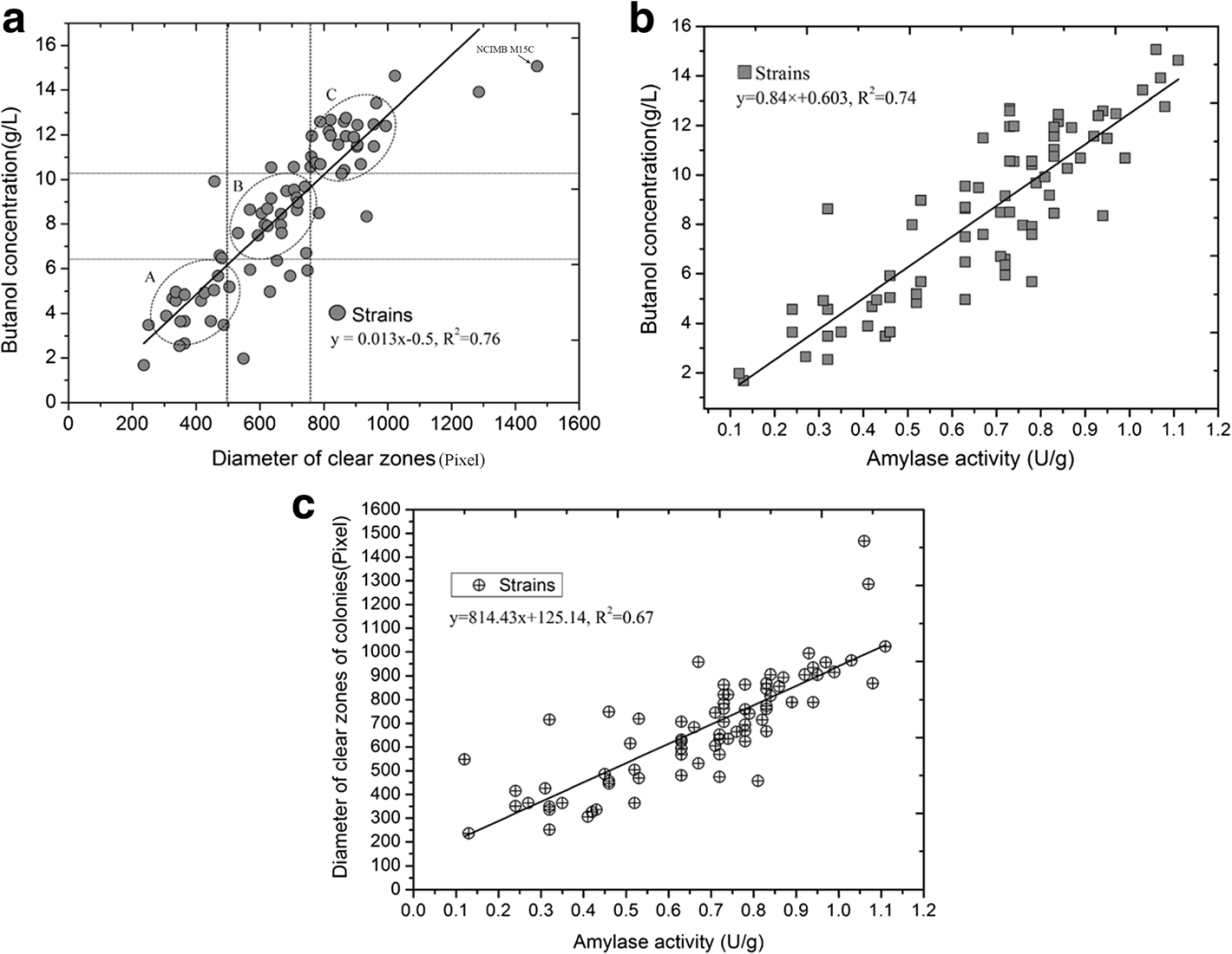
Investigation of the relationships between butanol production and diameters of clear zone and α-amylase activity. **a** The linear relationship between butanol production and diameters of clear zone. Strain’s scatters were distributed in three regions. **b** The linear relationship between butanol production and α-amylase activity. **c** The linear relationship between diameters of clear zone and α-amylase activity

The diameters of the clear zone surrounding colonies that appeared on plates were measured using the software Image-Pro Plus 6 (Media cybernetics, Silver Spring, MD, USA). Butanol production from soluble starch substrates and α-amylase activity of all the mutant strains were also determined. We compared the diameters of the clear zone of all colonies against their highest butanol titer. There was an approximately positive linear correlation between the highest butanol titer and the diameters of the clear zone around colonies (Fig. 2a or see Fig. 3a of Ref. [30]). In addition, we observed a linear correlation between butanol production and α-amylase activity (Fig. 2b). The diameter of the clear zone surrounding colonies also had a linear correlation with α-amylase activity (Fig. 2c). The coefficient of determination (R^2^) is an important indicator to determine whether there is a linear correlation between the independent and dependent variable. When analyzing the correlations between the clear zone and butanol, butanol and α-amylase activity, and the clear zone and α-amylase activity, the R^2^ values for these statistical analyses were 0.76, 0.74 and 0.67, respectively, suggesting that the correlations had a credible linear relationship. These results demonstrated that using soluble starch medium containing trypan blue dye to identify and screen target mutants with high butanol production from fermentation of starch was a feasible approach. This approach appears to enable easier identification of high-yield strains.

In addition, we observed that the regional distribution of strain’s scatter in the relationship between the diameter of clear zone and butanol production could be divided roughly into three regions: A (0 ~ 500), B (500 ~ 750), and C (750 ~ 1500) according to the scattered characteristics of bacterial colony (dotted lines) (Fig. 2a). The butanol production (mean), α-amylase activity, and diameter of the clear zone for each region were calculated and the results are shown in Additional file 1: Figure S1. The calculated indexes demonstrate different values for each region, in which C > B > A.

We also observed that different colonies had different brightness in the clear zones surrounding the colonies. We randomly selected 10 colonies and named: *C. beijerinckii* NCIMB M8A, *C. beijerinckii* NCIMB M17A, *C. beijerinckii* NCIMB M27A, *C. beijerinckii* NCIMB M31B, *C. beijerinckii* NCIMB M38B, *C. beijerinckii* NCIMB M47B, *C. beijerinckii* NCIMB M12C, *C. beijerinckii* NCIMB M34C, *C. beijerinckii* NCIMB M53C, and *C. beijerinckii* NCIMB M15C (Additional file 1: Table S1) from different regions to investigate possible relationships between the brightness of the these zones surrounding the colonies and the α-amylase activity of each of the colonies. The brightness of the clear zones of these colonies was roughly estimated by visual observation, and the clear zone around the original strain *C. beijerinckii* NCIMB 8052 was used as a control for comparison (Additional file 1: Table S2).

In order to verify whether the changes in butanol of the mutant strains were indeed resulted from the altered α-amylase activities, we further investigated the metabolic pathways that clostridium utilizes for decomposing starch. In order to metabolize starch to glucose, only three enzymes are necessary, among which α-amylase is the most important one for this metabolic process ^24.^ In order to assess the α-amylase activity as well as the butanol production by fermentation, we selected two mutant strains as representatives from the A, B and C regions, respectively (Additional file 1: Figure S2), and we observed that strains that did not have inhibition of α-amylase activity produced more butanol. Our results confirmed that α-amylase activity is the key factor for obtaining high butanol production in this process.

### Relationships between colonies’ areas, butanol production, α-amylase activities, and diameter of the clear zones

**Fig. 3:**
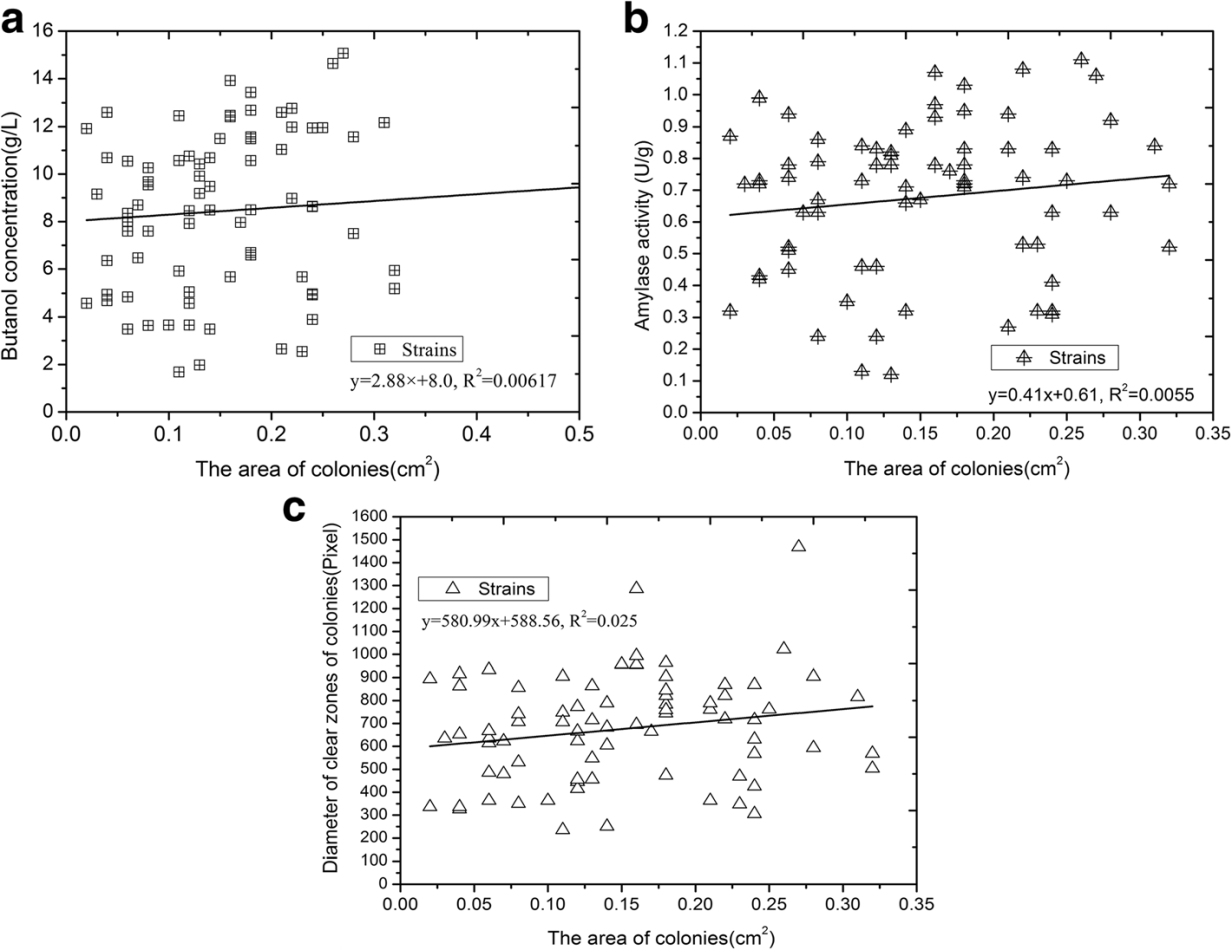
Relationships between colonies’ areas, butanol production, α-amylase activities, and diameter of the clear zones. **a** The linear relationship between butanol and colonies’ areas. **b** The linear relationship between colonies’ areas and α-amylase activity. **c** The linear relationship between diameters of clear zone and colonies’ areas

We observed that the colonies grew with areas of different sizes, so we further investigated the relationship between the colonies’ growth areas, butanol production, α-amylase activities, and the diameters of the clear zones surrounding the colonies (Fig. 3). First, we investigated the linear correlation between the butanol concentration and the area of colony growth. Based on the statistical results, there was no linear correlation between these two factors, because the R^2^ value (R^2^ = 0.0067) was beyond the scope of credibility (Fig. 3a). Second, we investigated the linear correlation between α-amylase activity and the colony areas was investigated. Similarly, there was also no significant linear correlation between α-amylase activity and colony area (R^2^ = 0.0055; Fig. 3b). Finally, we investigated the relationship between the diameter size of the clear zone and the size of the colony area. The results showed that there was no linear correlation between the clear zone diameter and area of the colonies (R^2^ = 0.025; Fig. 3c). Together, these statistical analyses demonstrated that there were no direct relationships between the size of colony area and butanol titer, α-amylase activity, and the diameter of the clear zone, which may be due to the competitive relationship between individual colonies in the microflora cluster, causing colonies with high amylase activity in colony intensive region have smaller colony diameters than those with low amylase activity in colony scarce regions.

### Fermentation characteristics of selected mutants

In butanol fermentation, genetic stability of strains is one of the important factors affect the efficiency of industrial fermentation. In addition, pH is also an important factor for improving butanol yield, making it necessary to investigate the the genetic stability effect under controlling pH. Representative mutant strains were randomly selected from the A, B, and C regions and used to ferment soluble starch; the fermentation characteristics were investigated in detail (Additional file 1: Figure S3). In order to determine the distinguishing fermentation features between mutant strains from different regions, we conducted batch fermentation in anaerobic bottles using soluble starch as substrate in order to quantify butanol titer. We compared the butanol titer from the mutant strains to those from the wild-type strains. Overall, we observed differences in butanol production between the mutant strains from A, B, and C regions. Mutant strains from the C region generated much higher levels of butanol (approximate 2-fold) compared to those from A and B regions (Additional file 1: Figure S3 a, Table 1). The representative mutant strains from the C region produced the highest butanol (13.76 g/L) and highest total solvents (22.39 g/L) in the least amount of time (96 h). The second highest butanol (10.23 g/L) was obtained from the mutant strains from the B region. In addition, more butanol was produced from the selected mutant strains compared with an average butanol concentration of 3.24 g/L produced by the wild-type *C. beijerinckii* NCIMB 8052 strain (Additional file 1: Figure S3 a).

**Table 1:** Performance (the highest value) of ABE fermentation products of selected mutants and the parental strain using soluble starch by batch fermentation

Parameters	8052	M8A	M17A	M27A	M31B	M38B	M47B	M12C	M34C	M53C
But. (g/L)	3.24^*a*^ ±0.45	5.12^*b*^ ±0.36	6.03 ^*b*^ ±0.27	5.23^*b*^ ±0.47	8.89^*c*^ ±0.39	9.26^*c*^ ±0.57	10.23^*c*^ ±0.62	13.76^*d*^ ±0.73	12.34^*d*^ ±0.69	13.46^*d*^ ± 0.85
Ace.(g/L)	1.11 ± 0.15	2.36 ± 0.34	2.11 ± 0.26	2.59 ± 0.43	3.68 ± 0.37	3.56 ± 0.28	3.51 ± 0.65	5.51 ± 0.43	5.11 ± 0.24	5.76 ± 0.37
Eth. (g/L)	0.58 ± 0.18	0.88 ± 0.25	1.12 ± 0.41	1.13 ± 0.31	1.52 ± 0.35	1.93 ± 0.31	1.68 ± 0.37	3.02 ± 0.45	3.33 ± 0.24	3.17 ± 0.53
Total ABE (g/L)	4.93	8.36	9.26	8.95	14.09	14.75	15.42	22.29	21.18	22.39
Total acids (g/L)	2.11	1.69	1.44	1.59	1.87	2.1	1.76	2.77	2.47	2.26
TS (g/L)	7.04	10.05	10.7	10.54	15.96	16.85	17.18	25.06	23.65	24.65
RCH (g/L)	30.72	14.32	20.44	18.44	11.34	12.34	13.22	7.62	7.17	8.47
ABE yield (g/g)	0.08	0.14	0.15	0.15	0.23	0.25	0.26	0.37	0.35	0.37
Product. (g/L.h)	0.05	0.08	0.10	0.09	0.15	0.15	0.16	0.23	0.22	0.23
But./TS(%)	46.02	50.95	56.35	49.62	55.70	54.63	59.54	54.90	52.17	54.60
FT(hours)	96	96	96	96	96		96	96	96	

*But* butanol (g/L), *Ace* acetone (g/L), *Eth* ethanol (g/L), *TS* total solvents (g/L), *RCH* residual carbohydrate (g/L), *Product* productivity (g/L.h), *But./TS* butanol/total solvents (%), *FT* fermentation time (hours)

ABE yield (g/g): total ABE production divided by gross weight of soluble starch. Productivity (g/L.h): Total solvents divided by fermentation time. ^*a,b,c.d*^: different letters in table indicate significant differences based on multiple comparisons (*P* < 0.05)

Mean values of three independent replicates each and standard deviations are shown

In terms of other metabolic products, mutant strains of the C region produced substantially higher levels of acetone than the mutant strains from other regions (Additional file 1: Figure S3 b and Table 1). The highest titer of acetone (5.76 g/L) and ethanol (3.33 g/L) were obtained from mutant strains from region C (Additional file 1: Figure S3 b and d and Table 1). The maximum titer of other compounds (butyrate and acetate) was likewise obtained from the mutant strains from region C in a period of 48 ~ 72 h (Additional file 1: Figure S3 d and e). Together, these results demonstrated that mutant strains with changes in metabolism leading to high α-amylase activity also had significantly enhanced butanol production.

We also observed that starch was not completely consumed by the mutant strains, and its consumption levels were the lowest in mutant strains from the A region compared to the mutants from regions B and C. For mutant strains from the A region, the residual amount of soluble starch for *C. beijerinckii* NCIMB M17A was up to 20 g/L, while for *C. beijerinckii* NCIMB M8A and *C. beijerinckii* NCIMB M27A the residual starch was only 14.32 g/L and 18.41 g/l, respectively. For strains from the B region, starch was more fully utilized, and the residue amounts were 11.34 g/L for *C. beijerinckii* NCIMB M31B, 12.34 g/L for *C. beijerinckii* NCIMB M38B, and 13.22 g/L for *C. beijerinckii* NCIMB M47B, respectively (Additional file 1: Figure S3). For mutants from region C, the total residual starch concentrations were the lowest with 7.62 g/L for *C. beijerinckii* NCIMB M12C, 7.17 g/L for *C. beijerinckii* NCIMB M34C, and 8.47 g/L for *C. beijerinckii* NCIMB M53C, respectively (Additional file 1: Figure S3). However, mutant strains selected from all regions consumed higher levels of soluble starch than the wild-type strain. The residual amount of soluble starch for the wild-type strain was 30.72 g/L. In total, compared to the wild-type strain, the mutant strains have higher α-amylase activity, which also led to elevated butanol production from the mutant strains compared to the wild-type strain. In addition, the growth of the strains has been quantified by measuring OD_600_, as shown in Additional file 1: Figure S3 g. The OD values showed that the mutant strains from the C region had the highest growth rates, which explains the hydrolysis levels of starch more fully.

The changes in pH during fermentation by the mutant strains and the wild type *C. beijerinckii* NCIMB 8052 strain are shown in Additional file 1: Figure S3 h. The largest pH change observed during fermentation was from mutant strains from region C (from 3.5 to 6.0; Additional file 1: Figure S3 h). At the end of fermentation, the pH values were above 4.0. The pH during fermentation by *C. beijerinckii* NCIMB 8052 stayed above 4.5 (Additional file 1: Figure S3 h). The changes in pH values reflected the total butanol titer; when the final pH was lower, the butanol titer may be higher. Mutant *C. beijerinckii* NCIMB M53C had a higher butanol titer at a final pH of 3.67 compared to that of *C. beijerinckii* NCIMB 8052 with a final pH of 5.5. These results also suggest that the efficiency with which the precursor (butyrate) was converted into butanol by mutant *C. beijerinckii* NCIMB M53C may be higher than the efficiency achieved by *C. beijerinckii* NCIMB 8052.

The highest butanol production was obtained by *C. beijerinckii* NCIMB M15C regardless of soluble starch (16.26 g/L) or glucose (20.67 g/L) (Additional file 1: Figure S4). Results showed that the bacterial strain with high amylase activity obtained high butanol titer (Additional file 1: Table S2 and Additional file 1: Figure S4). The α-amylase of *C. beijerinckii* NCIMB M15C was analyzed via homologous modeling (see Ref. [30]). The results showed that the highest butanol titer was obtained by *C. beijerinckii* NCIMB M15C, perhaps derived from the improvement of α-amylase activity of this strain. The results of α-amylase activities suggest that α-amylase is a pivotal factor for butanol production, probably because α-amylase produced mutation led to improvement of α-amylase activity, and thus increased butanol production. Therefore, feasibility of colony morphology analysis method based on trypan blue as a screened indicator rest with mutant strains present different α-amylase activity.

## Discussion

Although some screening methods have been used for screening target strains based on strain characteristics and colonial morphology, most of them are laborious and time-consuming, the success rate is very low [13–17]. For example, using strain tolerance to identity mutant strians were unsuccessful to date for obtaining high yield strains. In addition, other high-throughput screening method, for example, using detection agent to screen mutants can improve the success rate to a certain degree [25]. However, expensive diagnostic reagent kits is indispensable using this technique, it will greatly increase the cost of production.

In our work, we developed another method, first detailedly elucidated trypan blue as a screening indicator on a starch medium to identify target strains. This method has been investigated preliminarily by our previous study [30]. The results have showed the mutant strains with different cell viabilities via classic NTG mutagenesis can present different bright clear zones of the colonies and diameter of clear zone surrounding the colonies on trypan blue-dyed agar media. The availability of the method was comfirmed moderately based on limited data and analysis. Here, the method was further verified via assessing the bright clear zones of the colonies and specifically identify those with increased metabolic activities using more detailed data and statistical interpretation analysis. Our results demonstrated the applicability of this screening system. *C. beijerinckii* NCIMB 8052 was used to explore the relationship between the colony diameter of the mutant strains and the activity of α-amylase. Activity of α-amylase indirectly reflected the production of butanol. After chemical mutagenesis, a total of 76 clones were analyzed, and mutants with significantly increased butanol concentrations compared to the parental strain were selected. Using this method, mutant strains with high butanol production were more likely to be identified. Our statistical analysis confirmed the feasibility of this simple, intuitive and effective approach (Fig. 2). Further assessment showed that the effectiveness of this method was based on differences in the α-amylase activity of the mutants. Our results showed that mutants demonstrated the highest α-amylase activity also showed the highest butanol titer and the brightest clear zones. This screening method can bypass the physiological complexity of solventogenic clostridia to directly identify target strains. Due to multiple, largely unknown parameters that contribute to a particular phenotype, this screening strategy is useful for selecting a phenotype of interest because phenotypic differences can be visualized rapidly from a large population in mutant libraries.

**Fig. 4:**
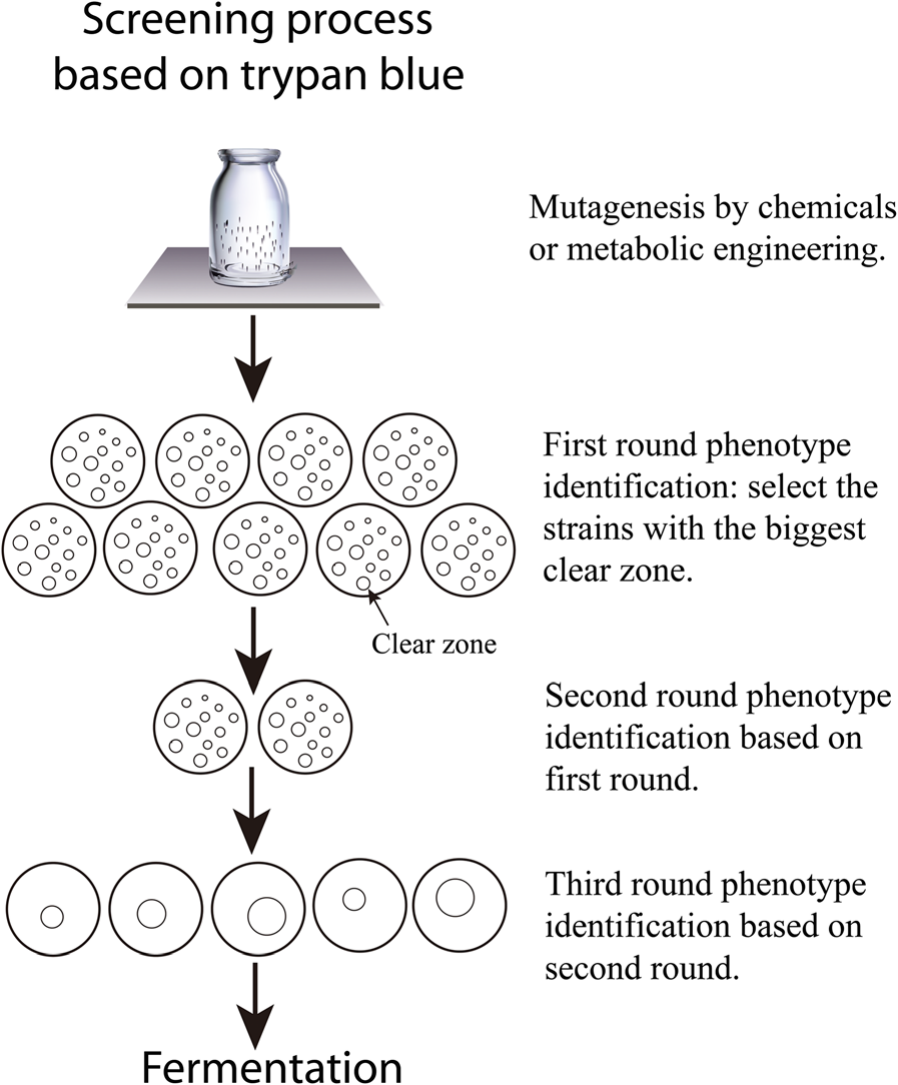
Flow sheet of the experimental design for screening processes based on starch medium with trypan blue

The screening procedure that we utilized was validated with three rounds of phenotypic characterization. In the first characterization, we pre-selected colonies with the largest diameter of clear zone, followed by a second screening where the identified mutant strains were transferred to a common culture dish, and the colonies with the largest diameter of clear zone were selected again. Finally, the mutant strains from the second selection were transferred one by one to the single medium to develop single colony, respectively, and the colonies with the largest diameter of clear zone were selected and tested for the capacity to produce butanol by fermenting starch (Fig. 4). The fermentation experiment also showed hereditary of mutant strain was stabilized under controlling pH value. Thus, the suitability of the visual, simple phenotypic characterization screening system was validated, generating up new approach for screening strategies to rapidly improve the selection of solventogenic clostridia and other biofuel microbes.

## Conclusions

**Fig. 5:**
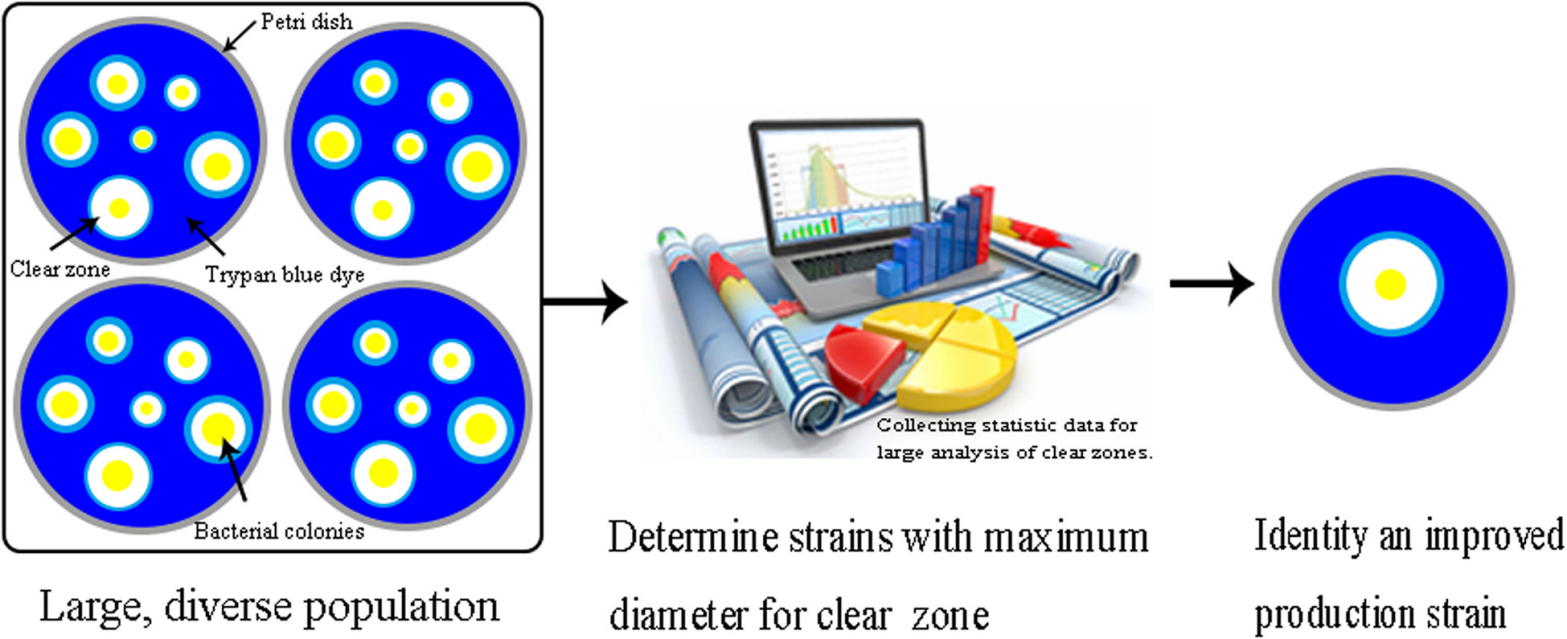
Presupposed and feasible screening scheme based on statistical analysis via collecting characteristic data

In conclusion, this study reported in detail about a simple, technically easy to operate screening method for biofuel microbes to specifically identify improved phenotypes based on α-amylase activity using trypan blue. The feasibility was demonstrated in a screening of clostridial mutant libraries and enhanced butanol production. The clones with high capacity were selected via collecting statistic data for the largest diameter of clear zone with graphics software (Fig. 5). This study found a correlation with a square error of 0.76 between the butanol production and diameter of clear zones. The screening method is fast, convenient, and reproducible and the technique can be easily adapted, enabling new screening strategies to optimize microbial biofuel production. In addition, the applicability can easily be broadened to a wide range of interesting microbes such as cellulolytic or acetogenic microorganisms, which produce biofuels from feedstock rich in starch.

## Abbreviations

ABE, acetone, butanol, and ethanol

## Additional file

Additional file 1: Figure S1. The mean butanol production, α-amylase activity, and diameter of the clear zone for each region were calculated. A, B and C indicate the strains’ scatter distribution. **Figure S2**. Inhibition of the α-amylase activities. (N) The α-amylase activity was not inhibited. (T) The α-amylase activity was inhibited. M17A, M27A, M31B, M38B, M12Cand M53C indicate selected mutant strains. **Figure S3**. Fermentation characteristics of selected mutants using soluble starch as fermentation substrate with controlling pH value. NCIMB M8A, NCIMB M17A, NCIMB M27A, NCIMB M31B, NCIMB M38B, NCIMB M47B, NCIMB M12C, NCIMB M34C, and NCIMB M53C indicate selected mutant strains. NCIMB 8052: wild type strain. **Figure S4**. Fermentation production of selected mutant strains based on different fermentation substrates. a: Butanol production of selected mutant strains from soluble starch and glucose. b: Change of ABE production of *C.beijerinckii* NCIMB15C from soluble starch and glucose. (PDF 2444 kb)
