# Supplementary material for: Investigation of availability of a high throughput screening method for predicting butanol solvent -producing ability of Clostridium beijerinckii
Source: BMC Microbiol. 2016 Jul 22;16:160. doi: 10.1186/s12866-016-0776-6 (PMC4957875; doi:10.1186/s12866-016-0776-6)
Supplement: Additional file 1: Figure S1. — The mean butanol production, α-amylase activity, and diameter of the clear zone for each region were calculated. A, B and C indicate the strains’ scatter distribution. Figure S2. Inhibition of the α-amylase activities. (N) The α-amylase activity was not inhibited. (T) The α-amylase activity was inhibited. M17A, M27A, M31B, M38B, M12Cand M53C indicate selected mutant strains. Figure S3. Fermentation characteristics of selected mutants using soluble starch as fermentation substrate with controlling pH value. NCIMB M8A, NCIMB M17A, NCIMB M27A, NCIMB M31B, NCIMB M38B, NCIMB M47B, NCIMB M12C, NCIMB M34C, and NCIMB M53C indicate selected mutant strains. NCIMB 8052: wild type strain. Figure S4. Fermentation production of selected mutant strains based on different fermentation substrates. a: Butanol production of selected mutant strains from soluble starch and glucose. b: Change of ABE production of C.beijerinckii NCIMB15C from soluble starch and glucose. (PDF 2444 kb) [file 12866_2016_776_MOESM1_ESM.pdf]

BMC MICROBIOLOGY

Development of a high throughput screening method for predicting the solvent-producing ability of Clostridium beijerinckii based on starch substrate

Haifeng Su[1] Jun Zhu[4] Gang Liu[3]* Furong Tan[2]*

*Correspondence: 350903876@qq.com; furong987@126.com
1: Enviromentally-Begnin Chemical Process Research Center, Division of Ecological & Enviromental Research on the Three Gorges, Chongqing Institute of Green and Interligent Technology, Chinese Academy of Science
2: Biogas Institute of Ministry of Agriculture, Chengdu, 610041, Sichuan, PR China
3: Sichuan Academy of Grassland Science, Xipu Chengdu, 611731, Sichuan, PR China
4: Rice Research Institute, Sichuan Agricultural University, 611130 Wenjiang, Sichuan, China

Table S1. Bacterial strains used in this study.

Strain	Relevant characteristics	Reference
C. beijerinckii NCIMB 8052	Wildtype	American type culture collection
C. beijerinckii NCIMB M8A	mutant of NCIMB 8052	this study
C. beijerinckii NCIMB M17A	mutant of NCIMB 8052	this study
C. beijerinckii NCIMB M27A	mutant of NCIMB 8052	this study
C. beijerinckii NCIMB M31B	mutant of NCIMB 8052	this study
C. beijerinckii NCIMB M38B	mutant of NCIMB 8052	this study
C. beijerinckii NCIMB M 47B	mutant of NCIMB 8052	this study
C. beijerinckii NCIMB M12C	mutant of NCIMB 8052	this study
C. beijerinckii NCIMB M34C	mutant of NCIMB 8052	this study
C. beijerinckii NCIMB M53C	mutant of NCIMB 8052	this study
C. beijerinckii NCIMB M15C	mutant of NCIMB 8052	this study

Table S2. Determination of α-amylase activities for selected mutant strains and original strains.Brightness of clear zones around colonies in STTY medium.

Strain	Total reducing sugar (g/L)	α-amylase activity (U/g)
C. beijerinckii NCIMB 8052	1.67 ±0.36	0.25 ±0.057
C. beijerinckii NCIMB M8A	2.45 ±0.22	0.36 ±0.063
C. beijerinckii NCIMB M17A	2.43 ±0.15	0.31 ±0.045
C. beijerinckii NCIMB M27A	2.28 ±0.14	0.41 ±0.059
C. beijerinckii NCIMB M31B	3.82 ±0.15	0.53 ±0.25
C. beijerinckii NCIMB M38B	3.59 ±0.26	0.48 ±0.033
C. beijerinckii NCIMB M47B	3.35 ±0.32	0.46 ±0.053
C. beijerinckii NCIMB M12C	4.33 ±0.25	0.56 ±0.045
C. beijerinckii NCIMB M34C	4.6 ±0.14	0.62 ±0.06
C. beijerinckii NCIMB M53C	4.42 ±0.18	0.58 ±0.065
C. beijerinckii NCIMB M15C	6.35 ±0.32	1.06 ±0.053

All values are means ±SD values within columns.

Figure legends of supporting information

Fig. S1 The mean butanol production, α-amylase activity, and diameter of the clear zone for each region were calculated. A, B and C indicate the strains' scatter distribution.

Fig. S2 Inhibition of the α-amylase activities. (N) The α-amylase activity was not inhibited. (T) The α-amylase activity was inhibited. M17A, M27A, M31B, M38B, M12Cand M53C indicate selected mutant strains.

Fig. S3 Fermentation characteristics of selected mutants using soluble starch as fermentation substrate with controlling pH value. NCIMB M8A, NCIMB M17A, NCIMB M27A, NCIMB M31B, NCIMB M38B, NCIMB M47B, NCIMB M12C, NCIMB M34C, and NCIMB M53C indicate selected mutant strains. NCIMB 8052: wild type strain.

Fig. S4 Fermentation production of selected mutant strains based on different fermentation substrates. a: Butanol production of selected mutant strains from soluble starch and glucose. b: Change of ABE production of *C.beijerinckii* NCIMB15C from soluble starch and glucose.

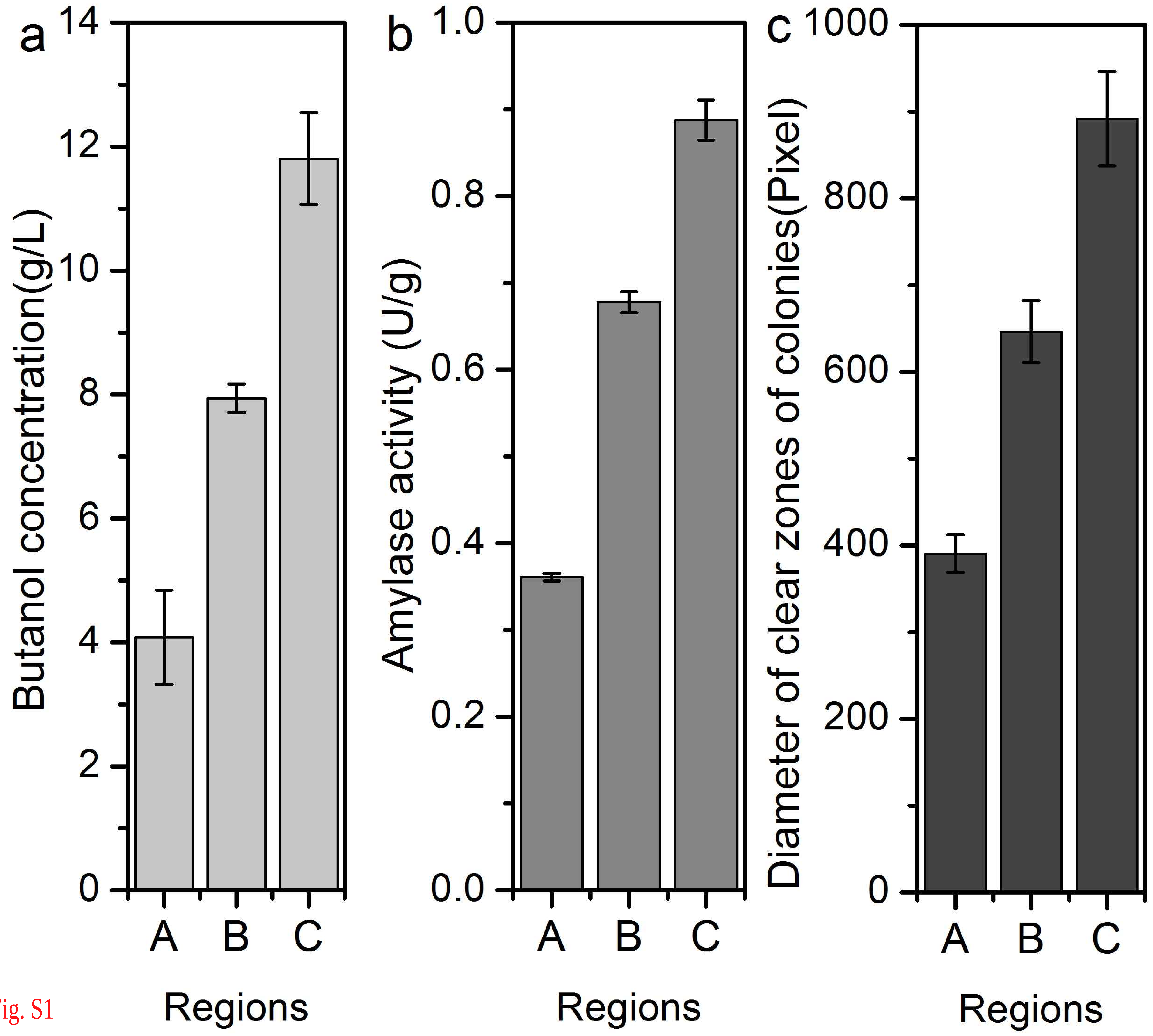

a
b
c
Butanol concentration(g/L)
Amylase activity (U/g)
Diameter of clear zones of colonies(Pixel)
0
2
4
6
8
10
12
14
0.0
0.2
0.4
0.6
0.8
1.0
0
200
400
600
800
1000
A
B
C
Regions

Fig. S1

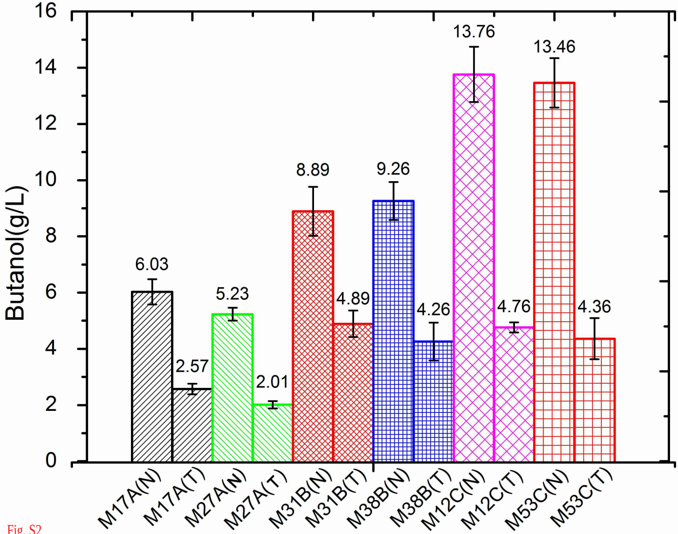

Butanol(g/L)
16
14
12
10
8
6
4
2
0
6.03
2.57
5.23
2.01
8.89
4.89
9.26
4.26
13.76
4.76
13.46
4.36
M17A(N)
M17A(T)
M27A(N)
M27A(T)
M31B(N)
M31B(T)
M38B(N)
M38B(T)
M12C(N)
M12C(T)
M53C(N)
M53C(T)

Fig. S2

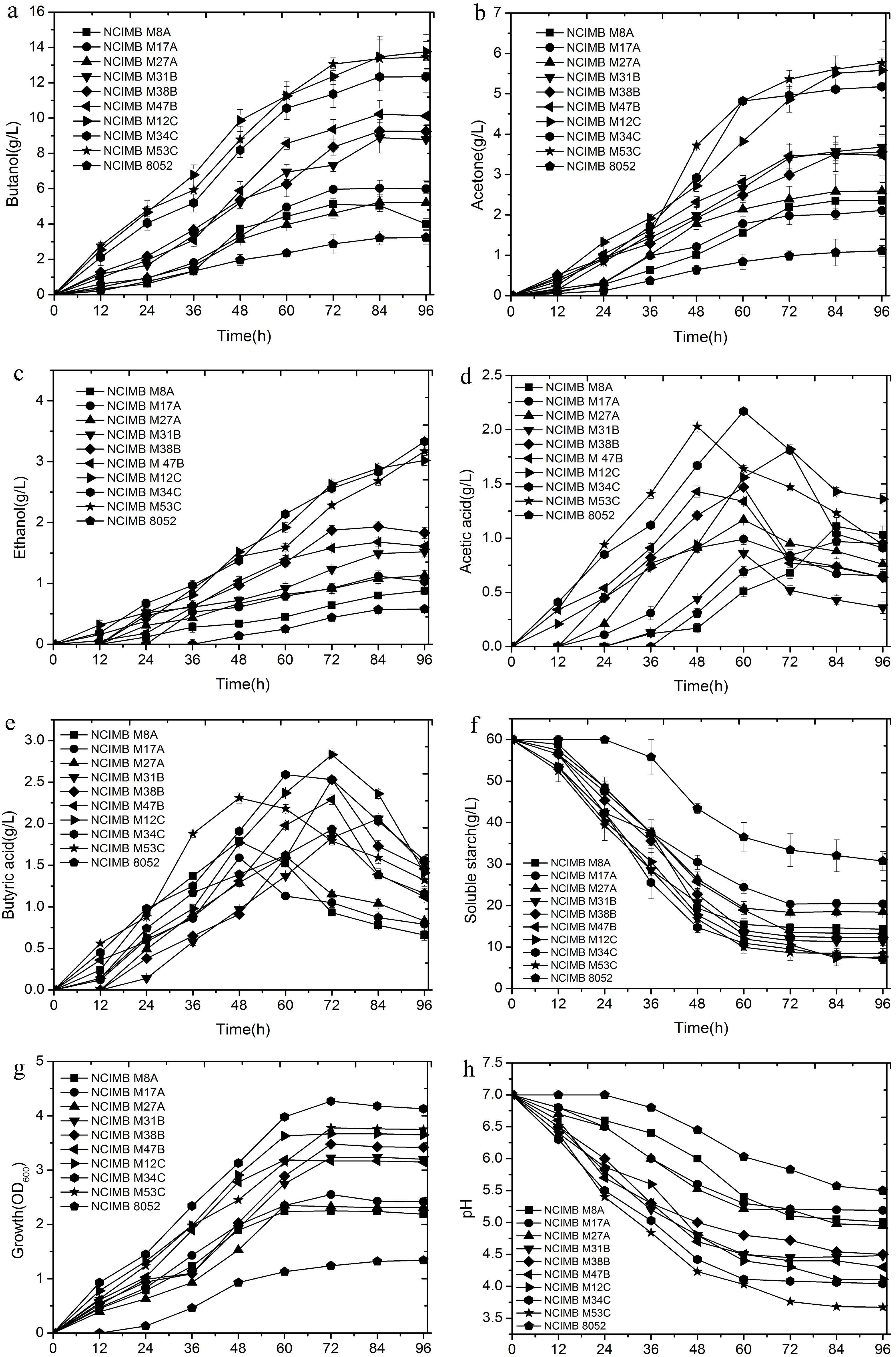
a
Butanol(g/L)
Time(h)
NCIMB M8A
NCIMB M17A
NCIMB M27A
NCIMB M31B
NCIMB M38B
NCIMB M47B
NCIMB M12C
NCIMB M34C
NCIMB M53C
NCIMB 8052
b
Acetone(g/L)
Time(h)
NCIMB M8A
NCIMB M17A
NCIMB M27A
NCIMB M31B
NCIMB M38B
NCIMB M47B
NCIMB M12C
NCIMB M34C
NCIMB M53C
NCIMB 8052
c
Ethanol(g/L)
Time(h)
NCIMB M8A
NCIMB M17A
NCIMB M27A
NCIMB M31B
NCIMB M38B
NCIMB M 47B
NCIMB M12C
NCIMB M34C
NCIMB M53C
NCIMB 8052
d
Acetic acid(g/L)
Time(h)
NCIMB M8A
NCIMB M17A
NCIMB M27A
NCIMB M31B
NCIMB M38B
NCIMB M 47B
NCIMB M12C
NCIMB M34C
NCIMB M53C
NCIMB 8052
e
Butyric acid(g/L)
Time(h)
NCIMB M8A
NCIMB M17A
NCIMB M27A
NCIMB M31B
NCIMB M38B
NCIMB M47B
NCIMB M12C
NCIMB M34C
NCIMB M53C
NCIMB 8052
f
Soluble starch(g/L)
Time(h)
NCIMB M8A
NCIMB M17A
NCIMB M27A
NCIMB M31B
NCIMB M38B
NCIMB M47B
NCIMB M12C
NCIMB M34C
NCIMB M53C
NCIMB 8052
g
Growth(OD600)
Time(h)
NCIMB M8A
NCIMB M17A
NCIMB M27A
NCIMB M31B
NCIMB M38B
NCIMB M47B
NCIMB M12C
NCIMB M34C
NCIMB M53C
NCIMB 8052
h
pH
Time (h)
NCIMB M8A
NCIMB M17A
NCIMB M27A
NCIMB M31B
NCIMB M38B
NCIMB M47B
NCIMB M12C
NCIMB M34C
NCIMB M53C
NCIMB 8052

Fig. S3

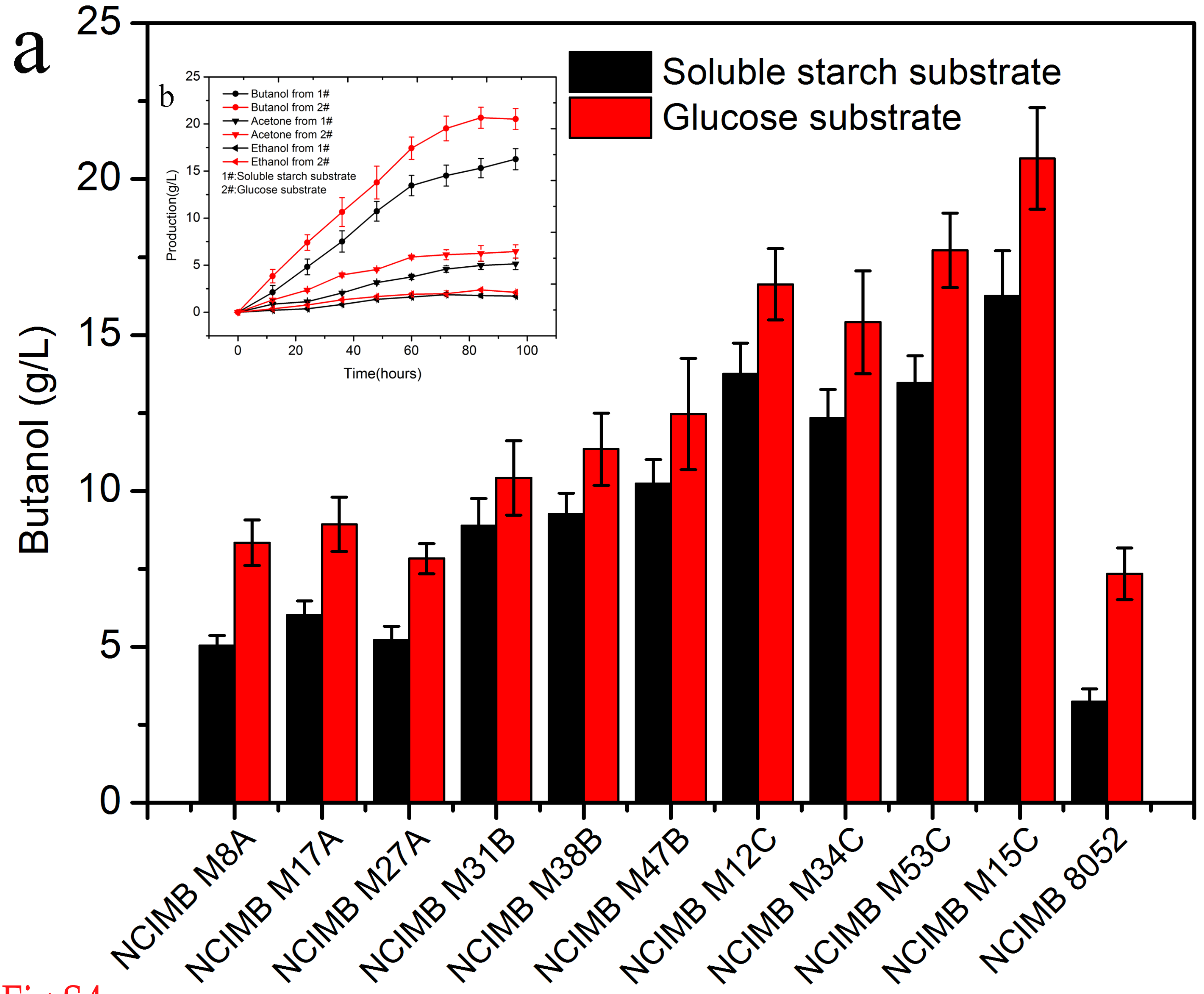

a
Soluble starch substrate
Glucose substrate
Butanol (g/L)
0
5
10
15
20
25
NCIMB M8A
NCIMB M17A
NCIMB M27A
NCIMB M31B
NCIMB M38B
NCIMB M47B
NCIMB M12C
NCIMB M34C
NCIMB M53C
NCIMB M15C
NCIMB 8052
b
Butanol from 1#
Butanol from 2#
Acetone from 1#
Acetone from 2#
Ethanol from 1#
Ethanol from 2#
1#:Soluble starch substrate
2#:Glucose substrate
Production(g/L)
Time(hours)
0
20
40
60
80
100

Fig.S4
